# The State of Learning Patterns within Medical Education in a Post-pandemic World: Reflection from IJMS Authors and an Overview of the IJMS Volume 10 Issue 3

**DOI:** 10.5195/ijms.2022.1695

**Published:** 2022-09-28

**Authors:** Ahmed Nahian, Richard Christian Suteja, Duha Shellah, Ciara Egan, Mihnea-Alexandru Găman, Francisco J. Bonilla-Escobar

**Affiliations:** 1 California Baptist University-Lake Erie College of Osteopathic Medicine, Riverside, CA, United States. Student Editor, IJMS.; 2 Udayana University - Faculty of Medicine, Denpasar, Bali, Indonesia. Student Editor, IJMS.; 3 Faculty of Medicine and Health Sciences، An-Najah National University، Nablus, Palestine. Junior Associate Editor, IJMS.; 4 Medical Student. Humanitas University, Humanitas Research Hospital, Milan, Italy. Deputy Editor, IJMS.; 5 MD, PhD student. Faculty of Medicine, “Carol Davila” University of Medicine and Pharmacy, 050474 Bucharest, Romania & Department of Hematology, Center of Hematology and Bone Marrow Transplantation, Fundeni Clinical Institute, 022328 Bucharest, Romania. Scientific Editor, IJMS.; 6 MD, MSc, PhD(c). Researcher, Department of Ophthalmology; Institute for Clinical Research Education (ICRE), University of Pittsburgh, Pittsburgh, PA, United States. CEO, Fundación Somos Ciencia al Servicio de la Comunidad, Fundación SCISCO/Science to Serve the Community Foundation, SCISCO Foundation, Cali, Colombia. Grupo de investigación en Visión y Salud Ocular, VISOC, Universidad del Valle, Cali, Colombia. Editor in Chief, IJMS.

The most defining aspect of medical education is the need for continuous and dynamic practice of the application of knowledge in the field and learning of how to communicate with and care for the patient. There is a fundamental need to connect the theory learned in the classroom with its experience and experimentation in real life; led by a teacher who challenges and guides the students on this path. This includes cadaver labs, histology slides and a microscope, simulation centers, skills labs, and clinical teaching rounds with patients. Critical to this learning is the repetitive nature of these experiences over time as well as a longitudinal relationship with a professor. In acknowledging this, where do we stand after two years of distance-learning that included pre-recorded lessons, online modules, communication through technology, and a halt to continuous, practical clinical learning?

In reaction to the COVID-19 pandemic, the American Association of Colleges of Osteopathic Medicine (AACOM) and Commission on Osteopathic College Accreditation (COCA) as well as the Association of American Medical Colleges (AAMC) and Liaison Committee on Medical Education (LCME) recommended delaying clinical rotations for medical students on March 17, 2020.^1^ Technologies such as telemedicine/telehealth emerged globally as mediums for interim medical education and alternatives to the conventional, in-person modes for training prospective doctors. This meant that in both 2020 and 2021, medical students in preclinical years had to adapt to learning practical skills from online modules, while those in their clinical years missed firsthand clinical skills due to enforced departure from their campuses. This adaptation served as a challenge, not only for institutions but also for medical students.

By now, medical students around the world have grown accustomed to this remote learning system and there have been positive and successful outcomes. Studies have shown that online learning was more satisfactory to participants than offline learning.^2,3^ In terms of medical knowledge, meta-analyses have found that online learning yields significantly positive academic outcomes.^4,5^ Creation of asynchronous classes and multiple way of reviewing a topic have enhance the students’ experience with flexible learning hours.^6^

**Shinjit Mani**, a medical student in his last year at the Kazan State Medical University, describes how the COVID-19 pandemic-related lockdown was implemented throughout Russia while researching the GIST430 cells’ resistance mechanism.^7^ Both his project and laboratory work were put on hold. There were just a few remaining crucial experiments and employees at the facility were working brief shifts. He could not work in the lab since visitors from outside the University were prohibited for security reasons. Shinjit had lectures and classes online and could only leave the dorm on weekends for three hours to acquire supplies. He began working on a separate project in the meantime that had higher statistical strength and could be completed quickly at home. His study correlating factors involved in colorectal cancer eventually found its place on the *International Journal of Medical Students (IJMS)*.^8^ Mani’s experiences fit the universal adaptations and adversities instigated by the pandemic.

**Azevedo Sansoni et al**., reports that most medical students in Italy were concerned about the fate of their surgical training during the pandemic. The cancellation of elective procedures resulted in shorter operating periods for surgical residents and early-career surgeons all around the world, which has decreased the opportunity for intraoperative practice and training.^9^ In Autumn 2020, the Master Surgeon Trust Collaborative group (UK) conducted an international survey that gathered data on 1604 eligible participants from 45 countries. According to 1305 of the participants (81.4%), the COVID-19 epidemic negatively impacted their training. Younger medical students, likely in their preclinical years, reported adverse effects on their comprehension of complex material. Medical students over 21 years old reported a higher volume of clinical responsibilities. These transformations opened up new challenges, behavioral and practical, to the medical profession at large.^10^

**Gardikioti et al**., share the experience of the Laboratory of Primary Health Care, General Practice, and Health Services Research on launching numerous student initiatives at the Medical School of the Aristotle University of Thessaloniki in Greece during COVID-19. Medical students in their fifth and sixth years showed exceptional drive and helped to form COVID-19 Reaction Teams, making them stand out from other Greek medical schools in terms of viewpoint and involvement. The student participation in every aspect of the initiative, including its inception, organization, implementation, and evaluation of the original aims, gave it a unique perspective.^11^

The COVID-19 pandemic also unfolds topics for scientific research, international collaborations and opportunities for funding. **Varma et al**., using secondary data from Europe, describe the trends and impacts of tick-borne encephalitis (TBE) in the region. There is a concern about the effects of global warming in emerging and reemerging diseases, and the authors make their argument about the increasing incidence of TBE and call for attention to this issue.^12^ At the *IJMS*, we are committed to science, global health, and the global warming threat to humanity, thus we will keep supporting preventive actions and calling out these situations as in previous editorials.^13–16^

In the Editorial by **Dable-Tupas et al**., the authors highlight opportunities for medical students including financial opportunities that medical students have to carry out research or present their results in different organizations and settings.^17^

**Pagador et al**., investigated the acceptance of COVID-19 vaccines among unvaccinated individuals residing in the Philippines. More than half of the 1011 surveyed subjects refused to be vaccinated due to concerns regarding safety or because they had no trust in vaccines. Essential healthcare workers, females, and practitioners of alternative medicine exposed to SARS-CoV-2-infected individuals were more likely to accept COVID-19 vaccines. In contrast, married subjects and persons employed as essential workers outside the healthcare system or in the private sector, or self-employed were not willing to accept vaccination.^18^ Similar studies should be carried out in other locations to build up evidence about barriers for vaccination and create strategies to enhance prevention.

The changes caused in everybody’s lives by the pandemic probably affected quality of life and how patients relate with the healthcare system. With regards of quality of life, **Haque et al**., sparked awareness about the differences in modular and annular curricula in medical school. Though, in the end, both curricula contain all the necessary knowledge to produce competent graduates, results showed that the modular system yielded higher quality of life for medical students compared to the annular system in some aspects of life.^19^ On the other side, **Ramdhany et al**., researched sleep quality among young people in Mauritius in the period between 2019–2020 and found that 30.7% of local youth reported sleeping poorly.^20^

The pandemic, however, has also opened new doors to interconnect peers and share the growth across the community. Over one-third of participants in a study by **Siah et al**., believed that engaging with a Facebook page for the Cardiff University School of Medicine boosted their satisfaction with the courses. The results also demonstrate that professional growth and interaction with medical professionals outside medical school are made possible by the Cardiff University C21 Facebook page.^21^

**Chandra et al**., shared the efforts of the preclinical medical student from Universitas Indonesia (FMUI) to empower the Indonesian community during the COVID-19 pandemic. The authors utilized online platforms to maximize engagement with the community and to build a good rapport by holding events in the *PrimaKu* app, an app of the Indonesian Pediatric Society designed to monitor child growth and development.^22^

In terms of changes in the healthcare system, **Jack Allen**, in a systematic review, used qualitative research to investigate the experiences of adults who have used remote consultations in primary care settings. Three themes were found, including advantages, potential disadvantages, and advantageous prerequisites for telemedicine consultations in primary care. The most frequently encountered hindrance to such consultations was a lack of face-to-face and physical interaction.^23^

Finally, in a more clinical setting, **Farris et al**., studied the blood vessels to raise awareness of variations in testicular arteries and promote the uniformity of preoperative vasculature investigation to reduce intraoperative risk to living male kidney donors and to improve patients’ understanding of potential risks and complications before consenting to the procedure.^24^

## Conclusion

As intuited earlier, medicine extends beyond memorization and books. Aside from technical barriers which affected the delivery of online classes, students were missing real-life experiences and character-building lessons that cannot be delivered online. These barriers are slowly perishing as a more adapted cohort of prepared medical students return to in-person learning. While some regions still require some form of social distancing or safety protocols, in-person classes are maximizing social interaction skills and empathy towards patients - an irreplaceable component of a competent physician.^6^ Community services, which once served as a good practice to grow medical students’ social spirit, have evolved into new forms, including online experiences.^7^ Pandemic-led limited interaction with faculty members that inhibited the sharing of knowledge and passion about medical research are now healing as well. These non-academic aspects of medical school are exactly why a fully online medical education course to procure highly competent doctors was not fully envisioned.

As the medical world is now shifting back to conventional offline teaching, students need to brace for what the future of medical education holds for them. Though medical students are more prone to burnout,^25^ which is especially true for new medical students who never experienced pre-pandemic offline learning, not all post-pandemic changes are negative, as discussed. Challenges and exciting opportunities are something to behold. Community services, clinical rotations, and student research activities may have transformed, but they exist to proliferate medical student experiences and skills.

As a diffusion platform for medical student research, the *International Journal of Medical Students (IJMS)* has worked tirelessly in recent months to reignite medical students’ passion for research by proposing the World Conference of Medical Student Research (WCMSR).^26^ The WCMSR will be the largest international medical conference with exclusive participation of medical students and recently graduated physicians from all over the world. This conference aims to show the world of medicine that students, too, are a major drive in the advancement of medical research. These present-medical students are the world’s future leaders in healthcare.

As the world gets back to a new, post-pandemic form, medical students across the world should understand the value of medical education on a practical scale and appreciate the gift of life to care, serve, and fight for it against all odds.
